# Arthroscopic Excision of Intra-Articular Hip Osteoid Osteoma: A Report of 2 Cases

**DOI:** 10.1155/2012/820501

**Published:** 2012-12-11

**Authors:** Alexandre H. Nehme, Alaa G. Bou Ghannam, Joseph P. Imad, Fouad C. Jabbour, Ramzi Moucharafieh, Joseph Wehbe

**Affiliations:** Department of Orthopedic Surgery and Traumatology, Saint Georges University Medical Center, Balamand University, P.O. Box 166378, Achrafieh, Beirut 1100 2807, Lebanon

## Abstract

Intra-articular osteoid osteoma is uncommon accounting for approximately 12% of all osteoid osteomas. It presents diagnostic and therapeutic challenges since several traumatic or degenerative pathologies of the joint can be simulated with delay in the diagnosis. We report the clinical, radiographic, and histopathological findings in 2 cases of intra-articular osteoid osteoma of the femoral neck and of the acetabulum. Technical aspects of arthroscopic excision and results of surgery are discussed. Arthroscopy allowed complete excision of the osteoid osteomas, with a short postoperative rehabilitation and excellent functional results.

## 1. Introduction

Osteoid osteoma is a benign osteoblastic tumor affecting young adults [1] and occurs preferentially in the shaft of long bones near the metaphyseal junctions [2].

Only 1% to 3% of all cases are located within the pelvis area [3], and when the hip joint is involved, there is usually an atypical presentation resulting frequently in delayed diagnosis compared with diagnosis of extra-articular osteoid osteoma [4].

Of the various surgical options including wide open [5] or CT-guided minimally invasive techniques [6, 7], arthroscopic excision was selected in our 2 reported patients for the following reasons.

1. The first patient had concomitant labral tear with an intra-articular osteoid osteoma of the base of the femoral neck that can both be simultaneously addressed by arthroscopy.
2. The second patient had the lesion located at the superior portion of the acetabulum, where CT-guided techniques might cause damage to the femoral head.

This case report discusses technical aspects of arthroscopic excision and results of surgery. The 2 lesions were preoperatively localized using 3D reconstruction software (Amira 5.2.0 from Visage Imaging) allowing for accurate targeting of the lesion. IRB/Ethics Committee decided approval was not required for this study.

## 2. Case Report 1

A 24-year-old male with no previous medical history presented to our clinic, complaining of progressive pain and restriction of motion of his right hip. Pain was located both in the pertrochanteric region and in the groin and was more severe at night.

**Figure 1:**
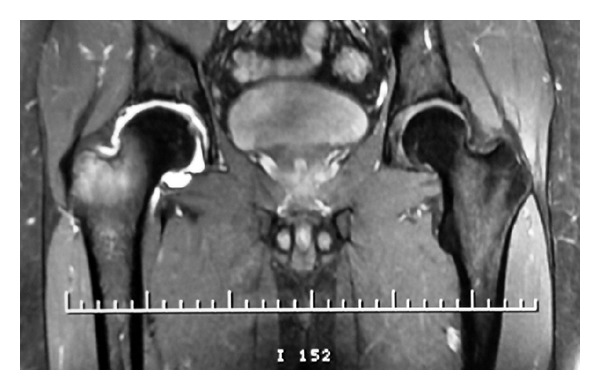
MR arthrogram of the right hip showing a lesion located at the base of the femoral neck.

On physical examination, pain increased with flexion of the hip and compression over the trochanteric bursa. Neurovascular examination of the extremity was normal. Plain radiographs of the pelvis and of the right hip showed no abnormality. Therefore, the patient was diagnosed to have trochanteric bursitis, and an injection of methylprednisolone acetate suspension (40 mg) was done in the pertrochanteric area. Symptoms were partially relieved for one week after this injection, and pain worsened afterwards especially during the night. An MRI of the right hip was done, confirmed the diagnosis of trochanteric bursitis without any visible additional abnormality in the intra-articular space. Conservative management was chosen, and 15 sessions of physiotherapy were done but with no improvement. Therefore, an MRI arthrogram was ordered to rule out any intra-articular pathology that might explain our patient's symptoms. It revealed the presence of a lesion located at the anterosuperior edge of the base of the femoral neck, approximately 0.5 cm in diameter, that displayed low-signal intensity both on spin echo (SE) T1-weighted and fast spin echo (FSE) T2-weighted magnetic resonance scans (Figure 1).

**Figure 2:**
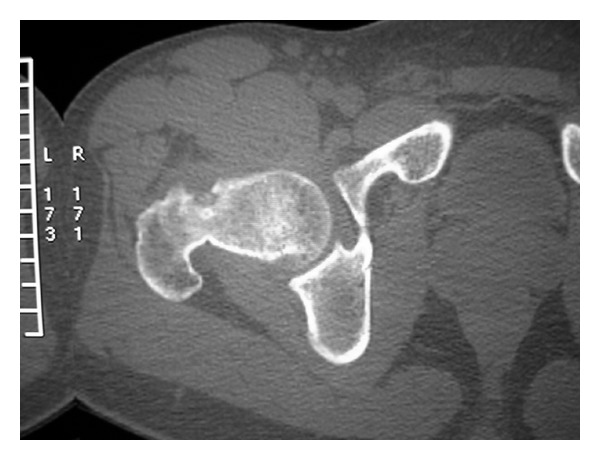
CT scan showing the subperiosteal nidus.

High resolution computerized tomography (CT) scans demonstrated the intraosseous location of subperiosteal nidus and the adjacent reactive medullary sclerosis with periosteal calcification (Figure 2). Radiographic results were compatible with the diagnosis of osteoid osteoma.

**Figure 3:**
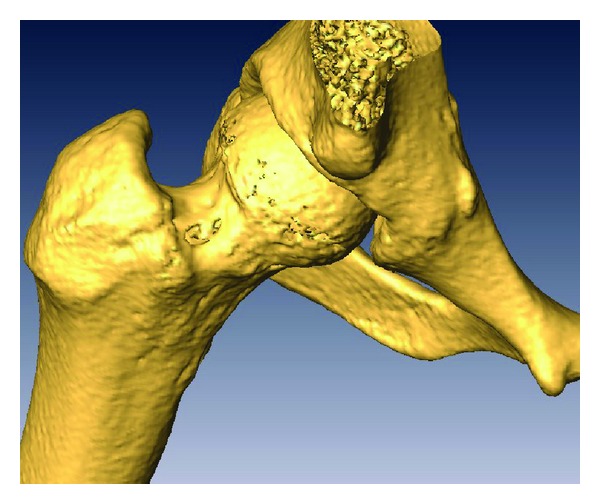
3D reconstruction of the proximal femur showing the lesion at the base of the neck.

3D reconstruction software (Amira 5.2.0) was used over the original stack of images obtained from the CT scan in order to generate a 3D model that facilitates accurate targeting of the lesion (Figure 3).

Hip arthroscopy was performed as follows, patient in supine position on a traction table under fluoroscopy guidance. We used 3 portals, the classic anterolateral for the camera, anterior, and anteroinferior for the instruments.

Exploration of the central compartment under traction was done to rule out any central pathology. There was a small radial flap and some fraying of the anterior sector of the labrum which was not detached. The flap and the fraying were resected using a 4.5 mm shaver. Traction was subsequently released, and the exploration of the peripheral compartment was achieved using the anterolateral superior and inferior portals. A capsulectomy was done using a 4.5 mm shaver blade and also a 4.0 resector (RF ablation system from Stryker).

Wide-spread synovial inflammation was noted in the joint, thus meticulous synovectomy was performed. A slight distortion of the cortex on the anterolateral part of the femoral neck was noticed compatible with radiographic location of the tumor. This distortion was thought to be overlying the cortex of the nidus, so the lesion was identified easily.

**Figure 4:**
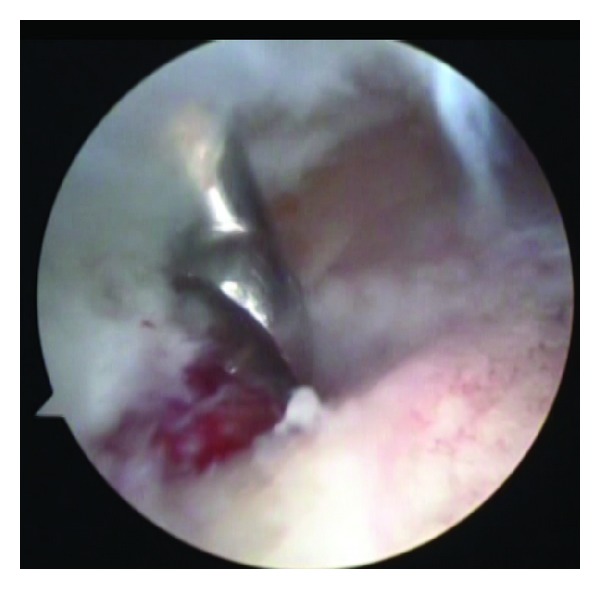
Resection of the nidus under arthroscopy.

Using an arthroscopic probe the overlying cortical bone segment was lifted from the underlying lesion. Thus, exposing a dark cherry-red nidus, with striking contrast in color to a background of normal cancellous bone. After visualization, the nidus was removed from its base, with the help of various sizes of straight and angled curettes (Figure 4). The fragile and loose specimens were removed from the joint carefully and gently using the grasper.

Bone base of the lesion was meticulously reexamined for identification of any residual tissue. Then bone surrounding the nidus was shaved using a round burr in order to make sure resection was complete. No prophylactic internal fixation and/or bone grafting for the residual defect was performed.

The histologic examination of the specimen showed the lesion to be an osteoid osteoma. During the postoperative period, an instant dramatic relief of pain was noted. The patient was kept nonweight bearing for 4 weeks. Partial weight bearing began after that. Full weight-bearing was allowed at the end of second month, without any problem. At the end of the first 24 months, the patient was still pain free, without any complaints.

## 3. Case Report 2

**Figure 5:**
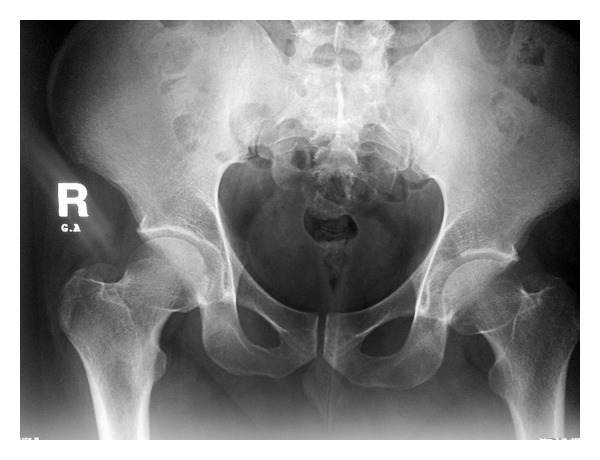
Ap Pelvis X-ray showing a lucency in the left acetabular dome.

**Figure 6:**
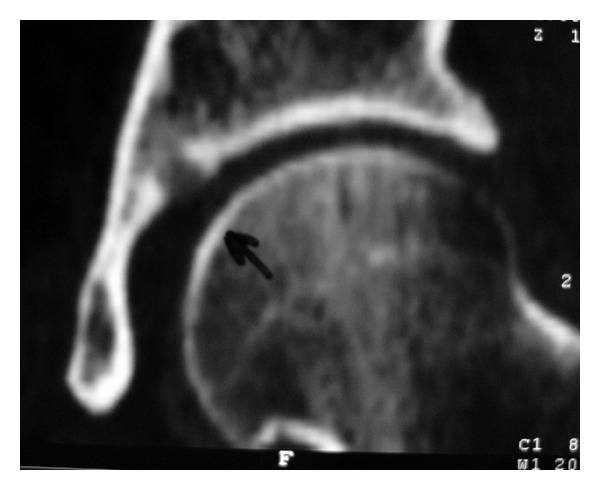
Coronal CT scan showing the lesion at the junction of the dome and notch of the acetabulum.

A 29-year-old male presented with a 12-month history of left groin pain. Pain exacerbates at night and was relieved with nonsteroidal anti-inflammatory drugs. On physical examination, pain was felt during maximal flexion and extension of the left hip. Anteroposterior pelvic radiograph showed a suspicious round lesion at the junction of the weight-bearing area of the acetabulum with the notch (Figure 5). Computed tomography showed a 0.9 cm sized sclerotic bony lesion within a circular lucency in the superior portion of the acetabulum (Figure 6). The radiographic diagnosis was osteoid osteoma.

Arthroscopic excision of the lesion was our choice of management in this particular anatomical location because CT-guided radiofrequency ablation would have caused excessive heat and damage to the acetabular and femoral head cartilage.

Hip arthroscopy was performed under general anesthesia using a traction table. The patient was put in the supine position, and the hip joint was distracted. The central compartment was approached using the anterior, anterolateral and anteroinferior portals.

Initial visualization, was a hip joint filled with blood clots and hyperemic synovium in the acetabular fossa. Blood clots were aspirated, and hyperemic synovium was removed with a motorized shaver. Localization of the lesion was done under the guidance of the scope and of the image intensifier. The nidus was removed using a curette, and, reactive sclerotic rim was removed using a motorized burr.

Total distraction time was 60 min. There were no resultant postoperative neurological complications. Lesion was confirmed to be an osteoid osteoma on histological examination of the curetted nidus.

The pain disappeared immediately after the operation. At 2-year followup, the patient was still symptom free with full range of motion of the hip joint.

## 4. Discussion

Intra-articular osteoid osteoma can be difficult to diagnose [2, 4]. Clinically, intra-articular osteoid osteoma may resemble traumatic or degenerative pathologies [2, 4]. Delay in diagnosis, therefore, may lead to muscle atrophy, tenderness, localized swelling, and possibly contractures [3]. Therefore, joint pain that is not responsive to conventional management needs a thorough diagnostic workup [5].

In our 2 case reports, the initial diagnosis was delayed approximately 6 months and 1 year and misdiagnosed as trochanteric bursitis and labrum injury. Initial plain radiographs were not diagnostic, and this experience has been previously described in the literature [1–4].

Conventional surgical approach requires a large incision, wide dissection, sometimes a hip dislocation, and considerable recovery time [5]. In the CT-guided ablation, destruction of the articular cartilage around the lesion is inevitable [6, 7]. Besides, the procedure is associated with a possible thermal damage and sometimes it is not possible to obtain a specimen for pathologic examination [6]. The advantages of arthroscopy are lesser surgical approach, accurate targeting and excision of the lesion, evaluation, and treatment of the possible resultant cartilage defect [8–10].

We conclude that the arthroscopic excision of the hip joint osteoid osteoma is effective because it causes minimal damage to normal bone or the adjacent cartilage, and because synovectomy for concomitant synovitis may be performed. Hip arthroscopy also yields a biopsy specimen adequate for pathologic examination.
